# Understanding stroke caregiving in rural contexts: a qualitative study of family caregivers’ cultural values, coping behaviors, and technology use

**DOI:** 10.3389/fneur.2026.1769482

**Published:** 2026-06-09

**Authors:** Mudasir Saleem Andrabi, Kunwal Scott, Betty Key, Kayla Lucena-Glass, Rylie Lancaster, Rebecca Martin, Robbin Young, Susan Appel

**Affiliations:** 1 The University of Alabama, Tuscaloosa, AL, United States; 2 Shelton State Community College, Tuscaloosa, AL, United States; 3 Northport Medical Center, Tuscaloosa, AL, United States; 4 The University of Alabama System, Tuscaloosa, AL, United States

**Keywords:** caregiver, chronic, rehabilitation, rural, stroke

## Abstract

**Background:**

Stroke is a leading cause of long-term disability worldwide, with recovery increasingly dependent on family caregivers who provide complex medical, rehabilitative, and emotional support. In rural and under-resourced settings, caregivers often assume these responsibilities with limited training and restricted access to formal services. Although caregiver burden is well documented, less is known about how cultural values, caregiving beliefs, and technology use shape caregiving practices and adaptation in real-world contexts where structural barriers are pervasive.

**Objective:**

To examine the experiences and support needs of rural family caregivers of individuals with chronic stroke, identifying culturally grounded caregiving behaviors, structural barriers to support, and technology-enabled strategies that can inform scalable, caregiver-centered interventions.

**Methods:**

Using a qualitative descriptive design, semi-structured interviews were conducted with 15 caregivers of rural-dwelling individuals with chronic stroke in Alabama. An integrative framework drawing on Leininger’s Transcultural Nursing Theory, the Health Belief Model, and the Technology Acceptance Model guided data collection and interpretation. Data were analyzed using Braun and Clarke’s six-phase thematic analysis.

**Results:**

Six interrelated themes emerged: cultural meaning of care and self-reliant caregiving; barriers to health services and limited formal support; informal learning and technology-based resources; family support and role adaptation; positive outlook on patient recovery; and emotional resilience through informal coping. Caregiving was framed as a moral and relational obligation rooted in cultural values, sustaining engagement despite emotional, physical, and financial strain. Caregivers reported barriers to formal support, including cost, insurance limitations, transportation challenges, and programs perceived as impractical. After brief in-hospital guidance, many relied on YouTube, social media, and telehealth to reinforce caregiving skills as needs evolved and professional support remained limited.

**Conclusion:**

Stroke caregiving in rural settings is shaped by cultural obligation, perceived necessity, and structural barriers. Caregivers function as extensions of the care team, highlighting the need for practical discharge training and accessible follow-up support. Culturally informed, family-centered, technology-enabled interventions that reduce cost and access burdens may improve caregiver well-being and strengthen stroke recovery outcomes.

## Introduction

Stroke survivors often require long-term, intensive, and highly specialized care (1). As health systems shift toward shorter inpatient stays and greater reliance on community-based management, family caregivers have become essential partners in recovery. Caregivers frequently supplement professional care by executing daily medical management, rehabilitation support, and assistance with activities of daily living (ADL) in home setting (2, 3). In chronic stroke, defined as the period beyond six months post-insult where initial neurological recovery has stabilized (4), the caregivers play a central role in the long-term recovery and prevention of secondary complications (5). Prior research has consistently documented of the profound burden experienced by the caregivers, including emotional distress, physical exhaustion, financial strain, and disruptions to employment and family life (6). These challenges are often compounded by limited access to ongoing professional support as care transitions into the home (7).

Despite these challenges access to structured caregiver education is inconsistent and often constrained by geographic, financial, and health system factors. Even when education is provided during acute hospitalization or rehabilitation, caregivers have difficulty retaining or applying information during high-stress post-discharge transitions (7). In practice, it is not uncommon for a spouse or parent caregiver to consult relatives, social media, or even YouTube videos for guidance on safely transferring a patient, managing feeding tubes, or preventing bedsores. In fact, online platforms like YouTube have become informal learning and support tools for caregivers, offering practical tutorials and a forum for hearing from others in similar situations (8). This pattern highlights not only caregivers’ adaptability and self-reliance, but also a significant system-level deficit wherein essential training and support are insufficient or inaccessible (9). The caregivers’ reliance on self-directed learning (3) reflects broader system-level gaps, including fragmented discharge processes and limited access to follow-up services (10).

Despite the increasing demands placed on the caregivers and the psychological stress associated with their roles (11), current research often fails to explore specific needs, preferences, and perspectives of caregivers living in resource-limited rural environments (11). Rural caregivers are particularly vulnerable due to transportation challenges, sparse local resources, and financial or insurance barriers (5). Existing programs and resources are not designed with the realities of low-resource environments in mind, leading to difficulty in accessing resources for rural caregivers (12).

Emerging evidence suggests that technology-driven psychosocial interventions can improve outcomes for caregivers. A recent systematic review and meta-analysis of stroke caregiving interventions found that digital approaches (e.g., telehealth programs, online education) significantly enhanced caregivers’ self-efficacy and caregiving competence, while also reducing anxiety and depression (13). These findings highlight the potential for technology to serve as a flexible, scalable mechanism for delivering caregiver education and emotional support across diverse settings. However, despite such promise, many interventions do not account for the realities of family caregivers in resource-limited environments.

To address these gaps, we conducted this study to better understand the needs, preferences, lived experiences of caregivers of rural-dwelling individuals with chronic stroke, with goal of informing the design of improved, contextually relevant support programs. Guided by an integrative conceptual framework on the health belief model (14), Leininger’s transcultural nursing theory (15), and the technology acceptance model (16), this study explores how caregivers learn and manage day-to-day caregiving tasks, navigate emotional and psychological demands, and engage with available resources within resource-constrained environments.

This study aims to examine the experiences of the caregivers of rural-dwelling stroke survivors through cultural, behavioral, and technological lenses, with particular attention to how cultural values shape caregiving meanings, how beliefs influence caregiving behaviors, and how technology is adopted to compensate for structural gaps in support. Understanding how caregivers learn complex tasks, cope with emotional demands, and engage with technology can inform the development of feasible, contextually relevant interventions.

## Methods

### Study design

This study employed qualitative descriptive design to explore the experiences, learning strategies, and support needs of family caregivers of individuals with chronic stroke. Semi-structured interviews were used for data collection to allow participants to describe their caregiver experience in their own words while ensuring all core topics were addressed. Data were analyzed using inductive thematic analysis guided by Braun and Clarke’s six-phase framework (17). This method allows for systemic identification and interpretation of patterns of themes across the data set. The study followed the Consolidated Criteria for Reporting Qualitative Research (COREQ) to ensure methodological transparency and rigor (18).

#### Conceptual framework

**Figure 1:**
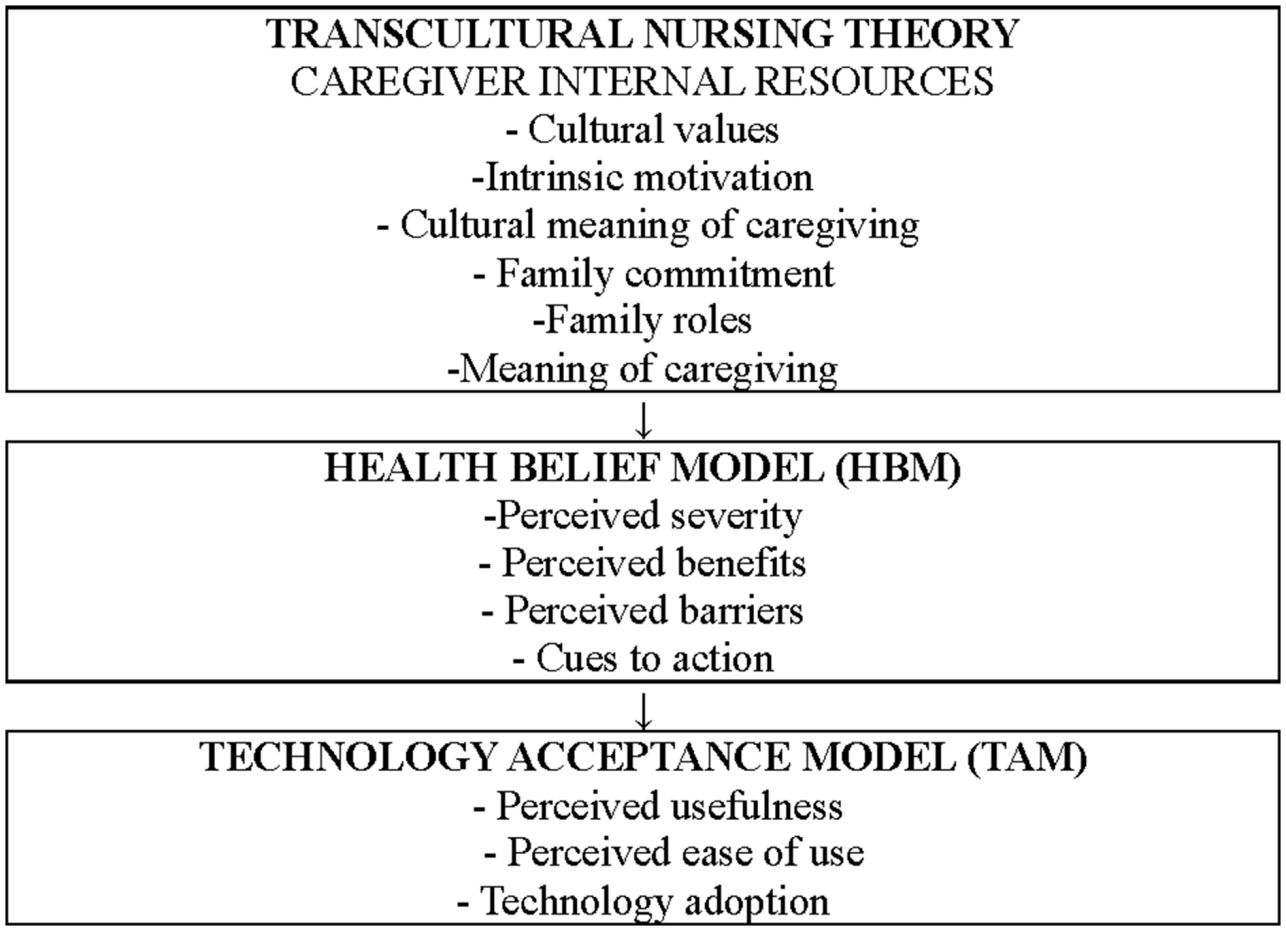
Conceptual framework: theory-guided understanding of stroke family caregiving.

This study was informed by an integrative conceptual framework drawing on Leininger’s Transcultural Nursing Theory, the Health Belief Model (HBM), and the Technology Acceptance Model (TAM) (Figure 1). Leininger’s Transcultural Nursing Theory guided the exploration of caregivers’ cultural values, family roles, and the meanings they attribute to caregiving, recognizing care as a culturally embedded practice. The Health Belief Model informed the examination of caregivers’ perceptions of stroke severity, perceived benefits of caregiving actions, and barriers to accessing formal support, which influence caregiving behaviors and coping strategies. The Technology Acceptance Model was used to understand caregivers’ adoption of digital and telehealth resources, emphasizing perceived usefulness and ease of use as key determinants of engagement. Together, these theories shaped the development of the interview guide, data interpretation, and thematic analysis by situating caregiving behaviors within cultural, behavioral, and technological contexts and enabling interpretation of how caregivers’ beliefs, cultural norms, and perceived barriers influence caregiving decisions and adaptation (See Figure 1).

### Participants and recruitment

Fifteen caregivers of rural-dwelling individuals with chronic stroke were recruited from an outpatient neurology clinic and from the community centers, including faith-based organizations and daycare centers. The Institutional Review Board (IRB) approved flyers were used to advertise participation in the study. Caregivers contacted the researchers using the phone numbers on the flyers. A telephonic screening identified the participants. Caregivers who were eligible and expressed interest were then met face-to-face by a member of the research team in a private area of the clinic or in a nearby faith-based organization (church), where they received a detailed explanation of the study and signed the informed consent. Eligible participants were ≥ 18 years old and identified as a primary or co-primary caregiver with at least a year of caregiver role for an individual with chronic stroke living in rural areas of Alabama. Purposive sampling was used to select participants. Out of 17 eligible caregivers approached, 2 declined to participate. Reasons for declining included lack of time due to caregiving responsibilities (*n* = 2). A total of 15 caregivers were interviewed (45 to 55 min) telephonically. Participants were required to provide information on ongoing caregiving support related to chronic stroke, including physical assistance, medical management, or support with daily activities. Participation was entirely voluntary, and caregivers were assured that declining or withdrawing would not affect the care received by the stroke survivor.

### Data collection

A semi-structured interview guide explored topics such as caregiving responsibilities, learning strategies, emotional coping, use of community resources, technology use to navigate the resources, and preferences for intervention programs. The semi-structured interview guide was pilot tested with two caregivers who met the inclusion criteria but were not included in the final sample. Minor revisions were made to improve clarity and flow of questions. The interview guide was reviewed by the IRB and the research team prior to data collection to ensure cultural sensitivity, minimize participant burden, and avoid potentially distressing or leading questions, reinforcing the study’s integrity.

Interviews were conducted via telephone. No family members or other individuals were present during the interviews unless specifically requested by the participant; in such cases, the presence of others was documented, and participants were reminded they could pause or reschedule to ensure privacy. Interviews were recorded and transcribed verbatim for analysis. Interview notes were taken during the interview. Follow-up interviews were conducted as needed for any clarifications. After the initial interview, two participants were contacted for another follow-up appointments for the clarifications related to their responses to the interview questions. Example interview questions included: “Can you describe how you learned to perform caregiving tasks?” “What challenges have you experienced in accessing caregiving support?” “What resources do you rely on when you need information or guidance?” and “How do technology or online tools support you in your caregiving role?”

### Data analysis

Braun and Clarke’s six-phase framework guided the inductive thematic analysis (17), which includes (1) familiarization with data, generating initial codes, (3) searching for themes, (4) reviewing themes, (5) defining and naming themes, and (6) writing the report. Two experienced qualitative researchers (MA, BK) independently engaged with the data, conducting thorough readings and repeated reviews of the transcripts. Data collection and analysis occurred concurrently to allow ongoing assessment of thematic saturation.

During this process, the researchers familiarized themselves with the data, highlighted key information, took detailed notes, and generated initial codes. These codes were then examined to explore connections and patterns, which informed the development of broader themes. Regular team meetings supported collaborative theme generation and review, aiming to reach consensus through comprehensive discussion and refinement. Saturation was considered achieved when no new codes, categories, or themes emerged in the final interviews, and existing themes were well developed and supported by multiple participants. After the 13th interview, no new themes were identified; two additional interviews were conducted to confirm thematic sufficiency. The research team reviewed the data iteratively and reached consensus that thematic saturation had been achieved. The authentic perspectives of caregivers of rural-dwelling stroke survivors were represented using direct quotations from data.

### Trustworthiness

Lincoln and Guba’s criteria for rigor and trustworthiness were used throughout the study. Verbatim transcription and independent analysis by two researchers ensured the Credibility. Sampling, data collection, and analysis were documented systematically to enhance dependability. Confirmability was maintained through ongoing review and the use of direct participant quotations. Transferability was supported by detailed descriptions of the study context, participants, and procedures. The interview guide was reviewed by an ethics expert and the research team prior to data collection to ensure that the questions were sensitive and relevant, reinforcing the study’s integrity.

Reflexivity was an ongoing process throughout the study. The interviewers had no prior personal or clinical relationships with the participating caregivers. The lead researcher, with experience in neurological rehabilitation, facilitated rapport while remaining mindful of potential assumptions related to clinical care. To minimize bias, the research team engaged in reflexive practices, including bracketing preconceptions, maintaining field notes documenting interviewer perspectives, and conducting regular team discussions to reflect on how researchers’ backgrounds and experiences might influence data collection and interpretation. Active listening and open-ended questioning were used to support participants in sharing their experiences in their own terms.

### Ethical considerations

IRB approval was obtained (IRB ID: 22-09-5982-R2) from the University of Alabama. The PI met with each participant in person at a location convenient to them. They were informed of the procedures in this study, and any questions or concerns were clarified with each participant individually. Participants were informed of the voluntary nature of participation and their right to withdraw at any time. Participants were offered rest periods during interviews to minimize participant fatigue and emotional burden. Each participant received a compensation of $25 gift card for their time following completion of the interview.

## Results

### Participant demographics

Participants included spouses, adult children, and other close relatives serving as primary caregivers. Several participants reported prior caregiving experience for family members with chronic illness, while others were new to the caregiving role. Most caregivers identified caregiving as consistent with family and cultural expectations of providing care for relatives during illness. The majority of participants, 14 (93%) were between 55 and 75 years of age, 13 (86%) had no college-level education, and 11 (73%) were living with stroke survivors. Ten (66%) participants were working, and 4 (26%) had children below 18 years in their household. Four (22%) participants had an annual income of below 10 k, 9 (60%) had it between 10 and 20 k, and 2 (13%) had 30- 40 k.

While the sample was relatively homogeneous in age and education, some variation in caregiving experiences was observed. For example, caregivers living with the stroke survivor described more continuous caregiving demands, whereas those living separately reported more episodic involvement. Additionally, caregivers with limited financial resources more frequently described barriers related to transportation and access to services.

### Thematic analysis results

The inductive thematic analysis identified six interrelated themes that reflect the reality of rural caregiving: internal strength and cultural resilience vs. external structural deficits. These themes include: (1) cultural meaning of care and self-reliant caregiving (19), limited access to formal support and barriers to health services, (3) informal learning and technology-based resources, (4) family support and role adaptation, (5) positive outlook on patient recovery, and (6) emotional resilience through informal coping strategies. These themes orient the reader to the caregivers’ role adoption to the development of informal coping and learning strategies.

#### Theme 1: cultural meaning of care, self-reliant and motivated caregiving

This theme was reflected by most participants, who described caregiving as a family responsibility rooted in cultural values and emotional commitment. The caregivers described a strong sense of independence in performing daily care tasks and emphasized learning through hands-on experience. Caregiving was portrayed not only as a responsibility but also as a meaningful opportunity to develop new skills. Care was frequently framed as an expression of love, duty, and relational commitment rather than as an imposed burden, with caregivers describing personal growth and fulfillment through their roles. This culturally grounded sense of responsibility reflects collectivist family norms and moral obligations embedded within caregivers’ social and cultural contexts, which sustained engagement despite emotional, physical, and financial challenges. Statements reflecting that caregiving was learned by doing demonstrate personal growth and confidence.

*P2: “My husband wants to try to do everything for himself at times, and he, you know, cannot do stuff for himself, and he gets agitated about it… frustrated about it like that, but I let him still try it and do. I try and learn too, he should do it too.”*

*P7: “Taking care of my mother was not very hard. I learned to do certain things by myself to take care of her. She was a little out of it, physically and mentally. But there was no one I had to take care of her. Once I got there, I calmed her down, told people to back off and give her space, and took care of her all by myself. She really thanked me for that because it did calm her down; she was having panic attacks for some reason.”*

Our results showed that caregivers often relied on intuition and trial and error when encountering unfamiliar tasks, underscoring self-reliance in the absence of structured training.

Intrinsic motivation emerged as a central driver of care. The caregivers consistently framed their efforts in terms of love and commitment for the person they cared for. For example, P6 mentioned that “did anything to help the person they care for.” This strong emotional foundation appeared to buffer challenges and sustain long-term engagement. Despite this motivation, the caregiver expressed a desire for clear, step-by-step instructions to guide specific care activities, indicating that self-reliance coexists with a need for concise, practical support.

#### Theme 2: barriers to health services and limited access to formal support

Nearly all participants described significant barriers to accessing formal support, including financial constraints, transportation challenges, and limited availability of services. Caregivers reported limited engagement with formal caregiving services, primarily due to financial and insurance barriers. Programs were often viewed as inaccessible or irrelevant, and the lack of insurance coverage for caregiving assistance was repeatedly described as a major constraint. These structural challenges curtailed opportunities for professional guidance and contributed to reliance on informal sources.

*P6 mentioned that “The barriers that influence the post-stroke care for my mother’s discharge after being at home could be things like a lack of transportation, limited insurance resources. No one to take care of my mother professionally. We sometimes cannot go to doctor appointments because the clinic is far from here. Sometimes, no one is there to take her to the doctor. Lack of resources would also mean no one to ask if we have any questions about her treatment. I don’t have anyone whom I can ask, but I do some small research on how to treat a stroke victim, but I didn’t find anyone in particular who can give me this knowledge. Facilitators influence post-stroke care after discharge to home, including the availability of smartphones for delivering education and providing motivation to learn”.*

*P1: “So, you know, some part of the family is out in cities making a living and things. So, we are here in the rural area with the puppy dogs and chickens. But our family is in this very rural area, so we have to take care of each other. Help cannot reach us. Um. My greatest, greatest success is seeing him smile. Okay. like once he finally achieved something that he really wanted to do, like, he was determined to search for dogs in the woods.”*

Financial considerations played a central role in determining which programs were perceived as feasible. The caregivers valued services that could “work with you without the payment,” suggesting that affordability and flexible payment structures would increase willingness to participate. These findings highlight significant gaps in the accessibility of caregiver support within the current healthcare landscape, particularly for individuals navigating neurological conditions. These challenges appeared more pronounced among caregivers with lower income and limited access to transportation, highlighting how resource constraints shaped access to formal support.

#### Theme 3: informal learning and technology-based resources

Most participants reported relying on digital platforms such as YouTube, Google, and social media to learn caregiving tasks and supplement limited formal training. Following initial guidance from nurses during hospitalization, caregivers reported relying on technology-based resources to supplement their caregiving knowledge once they transitioned to home care with limited formal support. Online tools, especially YouTube and social media, were described as primary sources for learning practical techniques. These platforms were perceived as accessible, easy to understand, and directly applicable to real-world caregiving situations. The caregivers also expressed confidence in their ability to prepare for future medical events by studying videos, underscoring the value of informal, multimedia learning.

P9 mentioned, *“Normally, when I want to know about how to take care of my husband who has problems due to stroke, I would just go on YouTube and Google and just find, you know, general knowledge like that. With those, really, you can find tutorials on how to handle people. Just how informative they are, I do not know, without the information, a person does not know what to do. So, I do search on YouTube Google, and you tube how to take care of my husband.”*

*P1: “Looking on the internet would make it easier if the information were out there in more than one way; you’d have more than one avenue to go. To find out more information about his condition, this is a good way to help those who have had a stroke.”*

Caregivers viewed technology-based interventions as especially advantageous due to transportation difficulties and scheduling constraints. Virtual care options, telehealth visits, video-based modules, and remote support groups were repeatedly identified as more realistic than in-person programs. This preference reflects both logistical limitations and the caregivers comfort navigating digital environments.

#### Theme 4: family support and role adaptation

Majority of participants expressed optimism regarding patient recovery, often describing small improvements as meaningful progress. Although formal support was limited, the caregivers benefited from intermittent assistance from friends and family members. The friends and family members or provided both emotional reassurance and occasional practical help with care tasks. Guidance received from nurses during the care recipient’s hospitalization also appeared to shape the caregivers’ early confidence and understanding of the role.

*As mentioned by P14: “Post-stroke is normally after the fact. My mother has had strokes in the past, and I normally have to just be a caregiver, you know, sometimes I have to rush to the hospital. Sometimes I have to cook for after the fact, you know, do household work, do small errands, and care about my mom too; but that’s what we do for our family, mainly just caregiving to her. I cannot leave her as she is my mom.”*

*P4: A lot of stuff I put to the side, to get to my mother and make sure she was okay, make sure she got to her apartment… and everything that I wanted to do. I could not do enough, because I live a bit far, and someone had to be there. I had two younger siblings, so they were young because there’s a 20-year age difference between us. They were not able to take care of her; they did not know what to do. I would come and sit… You know, doing things, spending the evenings with her. And on weekends when I was off… you know, I would have to just keep a watch out for her overnight.*

As survivors transitioned to the chronic stage, caregivers noted that care needs evolved and required ongoing modification, often with minimal professional support. In this context, family and friends’ involvement helped mitigate the absence of broader support structures and facilitated adaptation to new responsibilities. These informal support systems played an important role in sustaining caregivers and enhancing their ability to manage day-to-day care. The availability and extent of family support varied across participants, with some caregivers describing shared responsibilities, while others reported managing caregiving largely independently.

#### Theme 5: positive outlook on patient recovery

Many participants expressed a consistently optimistic view of the care recipients’ recovery trajectory, noting improvements in independence and functional ability. These observations were described with pride and appeared to reinforce the caregivers’ commitment to continued support. *For example, P3 mentioned, I would like to say that even though the effects of stroke are there, I believe that he would recover fully. I mean. It’s been a while, and it takes a while to get back what you lost. I do not know, I mean she should make it, if not 100%, maybe 80/90 percent.”*

*P2: “You just got to stay strong, as long as you stay strong, you’ll be alright. Don’t let anything get to you as I do sometimes because they’re in bed and can’t get up and be stuck and stuff like you just got to not let it go. When they have a bad mood, you just got to figure out a way to improvise to get them moving. Sometimes, a bad mood can lead to stress. You don’t want that on a person, trying to get their stuff back together because they’re already stressing enough. They’re going to need help with whatever they’re going through.”*

The recognition of recovery milestones contributed to a sense of progress and mutual accomplishment, which served as an emotional motivator. This positive orientation may play a protective role in caregivers’ well-being, as it frames challenges within a broader narrative of improvement and resilience.

#### Theme 6: emotional resilience and simple coping strategies

The caregivers reported some emotional distress and described relying largely on informal coping strategies, such as going for a social event or speaking with a friend when support was needed.

*P2: “There was so much stuff going on, like through it, I would have problems. I had car problems at one point in time, then I lost my job due to a stroke. It really just becomes a battle that you have to uphold. If you get it, just try to be responsible. Now taking care of my husband who had stroke. I don’t really go to many people, because I thought a stroke just slows you down, and you have to empower yourself to get back going with it. The church people supported us. That’s about it. When they come over here and pray, that gives you encouragement. Cause they give you another step and encouragement, I really do see it.”*

*P5: “I’ve been exercising and stuff like that, though! Sometimes I can ask for help. Sometimes I have a plan for my day. Right. What do I have to do? Sometimes I have to spend time by myself, and I have to plan it out.”*

*P12 mentioned that “I just talk to a friend when I am stressed or upset, and then, I just kind of like meditate a little bit to calm down, or go for a little walk, you know, I come back and everything was fine.”*

Although effective for immediate reassurance, the reliance on unstructured coping suggests a potential gap in formal emotional support tailored to caregivers of individuals with neurological conditions. This theme highlights opportunities to proactively address caregivers’ emotional health in an accessible manner (Table 1).

**Table 1:** Summary of major themes from caregivers’ interviews *N* = 15.

Theme	Description	Representative excerpts	Implications
Cultural meaning of care, self-reliant and motivated caregiving	The caregivers demonstrate autonomous engagement with care tasks, emphasizing independence, experiential learning and a profound motivation and commitment rooted in affective bonds.	“We take care of each other in a family.”	Suggests the need for concise, pragmatic training materials that augment intrinsic caregiver efficacy.
Barriers to health services and limited access to formal support	Structural impediments including inadequate insurance coverage and high costs substantially limit access to professional caregiving resources. Need for the support to overcome the barriers	“Lack of insurance to pay for a caregivers.” “lack of programs that work with you with the payment.”	Highlights critical gaps in systemic support necessitating affordable, flexible programmatic solutions.
Informal learning and technology-based resources	In the absence of formal education, caregivers utilize digital platforms such as YouTube and social media to acquire practical knowledge, facilitated by technological	“Basically I just rely on videos on YouTube.”/“Sometimes you do not have transportation to go to the provider office to get that care and knowledge.”	Endorses the development and integration of accessible, technology-mediated educational interventions tailored to caregiver needs.
Family support and role adaptation	Familial relationships, particularly with immediate relatives, provide essential emotional sustenance and instrumental assistance, compensating for limited formal services.	“It was my mom, I had to care for her. We care for our family and there was no other help.”	Emphasizes the necessity of incorporating family dynamics into caregiver support frameworks.
Positive outlook on patient recovery	Positive perceptions of the care recipient’s functional gains bolster caregivers’ perseverance and emotional well-being	She’s able to do for herself… needs help sometimes.”	Suggesting that supportive resources/ interventions that reinforce caregiver recognition of recovery milestones may enhance resilience and engagement.
Emotional resilience and simple coping	The caregiver maintains psychological equilibrium through informal strategies, such as social conversations, despite limited formal mental health support.	“I relieve my stress by just talking to a friend. We all go talk to family and friends when we need to”	Indicates an opportunity for targeted psychosocial interventions to complement existing informal coping mechanisms.

## Discussion

This study highlights a dual reality for family caregivers of stroke survivors that is directly grounded in participant accounts. Many caregivers described resilience and adaptive coping, although experiences varied across participants (“we have to take care of each other”) and a commitment driven by emotional bonds. At the same time, participants explicitly described structural barriers, including transportation challenges, financial constraints, and limited access to professional guidance. These findings demonstrate that caregiving practices are shaped by the interaction of culturally grounded values and constrained access to formal support.

Caregivers described learning caregiving skills through observation and guidance received from healthcare professionals during the in-hospital phase, and ongoing informal self-directed learning after discharge. Deficits, as reflected in participant accounts, many caregivers relied heavily on digital platforms such as YouTube and social media online searches to perform daily caregiving tasks in the absence of accessible professional support. These patterns align with established behavioral and transcultural nursing theories, suggesting that caregiving behaviors are shaped by cultural values, perceived necessity, and the accessibility of enabling resources rather than formal instruction alone. At the same time, participants reported persistent barriers, including transportation difficulties, financial constraints, and limited access to follow-up care. These findings indicate that caregiving practices are shaped by both culturally grounded responsibility and the necessity to adapt to gaps in formal support systems. Together, these findings add to existing literature by illustrating how caregivers in this study relied on self-directed strategies and available resources within their specific contexts, while emphasizing the need for accessible, technology-based, family-centered interventions. These results underscore an urgent need to bridge the gap between caregivers’ commitment and the external resources available to them.

Some variation in experiences was observed based on contextual factors. Caregivers with fewer financial and transportation resources described greater barriers to accessing formal support, while those living with the stroke survivor reported more continuous caregiving demands. These differences, although not systematically compared, provide important context for understanding variability in caregiver experiences.

Family caregivers of stroke survivors demonstrated remarkable resilience, intrinsic motivation, and adaptive coping strategies as reflected in participant narratives describing caregiving as a responsibility rooted in family obligation and emotional commitment. Many found personal growth and meaning in caregiving, framing tasks as fulfilling, and were driven by love and duty. These findings strongly reflect Leininger’s Theory of Transcultural Nursing, which posits that care practices are deeply embedded in cultural values, family roles, and lifeways (20, 21).

In this study, caregiving functioned not only as a health-related responsibility but as a culturally mediated expression of familial obligation and reciprocity, as illustrated by participants describing caregiving as something they “have to do” for family members, reinforcing persistence even in the absence of formal support.

Similar findings were in evidence from caregivers of dementia and Alzheimer’s disease patients, where acceptance and commitment support sustained engagement despite burden (22). Informal coping strategies such as positive thinking, prayer, and talking with friends mirrored findings in traumatic brain injury caregivers, who often manage stress independently (23). Optimism about patient recovery, including pride in minor improvements, parallels attitudes in Parkinson’s disease caregiving (24). Caregivers’ decisions to seek information, persist in caregiving tasks, and adopt coping strategies reflect core HBM constructs, including perceived severity, perceived benefits of caregiving actions, and perceived barriers to accessing formal services. From a HBM perspective, such optimism may function as a perceived benefit that reinforces continued caregiving behaviors despite perceived barriers (25). Importantly, these interpretations are grounded in participant accounts demonstrating how caregivers act despite limited formal support. These strengths highlight potential assets that interventions could leverage to support stroke caregivers.

A prominent finding grounded in participant narratives, was caregivers’ reliance on informal learning through digital platforms. Caregivers explicitly described using YouTube videos, social media groups, and internet searches. This reliance reflects both perceived usefulness and necessity in contexts where formal education and follow-up support were limited. This behavior is consistent with the HBM, wherein caregivers’ perceived severity of the patient’s condition and perceived responsibility motivate action, even when formal cues to action from healthcare systems are limited (25, 26). They valued these platforms for their accessibility and convenience, a trend increasingly observed among caregivers of cancer and Alzheimer’s disease (27).

Our participants expressed their interest and need for telehealth and remote communication with healthcare providers whenever possible. Several caregivers preferred virtual visits or phone consultations to overcome transportation barriers, as described in participant accounts highlighting distance and access challenges. Consistent with recent studies emphasizing the feasibility of telehealth for caregiver support in rural or resource-limited contexts.

The critical role of family support found here aligns with prior research in caregiving for chronic neurological disorders including stroke (28), dementia, and cancer, where family networks compensate for limited formal services (29). Within the Transcultural Nursing framework, family involvement is central to culturally congruent care, as family structures often function as primary care systems, particularly in under-resourced settings (30). Participants described relying on family members for both emotional and practical support, underscoring the importance of incorporating family dynamics into interventions. Family-centered approaches are likely to enhance caregiving sustainability by aligning with cultural norms of shared responsibility.

Financial, logistical, and programmatic barriers restricting formal support access have been documented among caregivers of cancer, Alzheimer’s disease, and neurological impairments (5, 24). The specific challenges faced by stroke caregivers in this study include insurance gaps and transportation difficulties, long distances to clinics, especially in rural areas, and inflexible program schedules that conflict with work or caregiving duties. From a HBM perspective, these barriers significantly reduce the likelihood of engaging with formal services, even when caregivers recognize their potential benefits (26). This reflects broader systemic inequities common to caregiving across chronic conditions. However, this study uniquely contextualizes these barriers within stroke care in rural areas, showing how caregivers innovate through informal and digital strategies when formal support is inaccessible or misaligned with their needs.

Our data also suggest that caregivers sometimes perceive available programs as irrelevant or unresponsive to their practical needs. For example, a caregiver dismissed a support class because it was too general and not hands-on enough. This highlights a mismatch between caregiver needs and formal service delivery. These findings indicate that caregivers rely on self-learning and family support out of necessity rather than preference. Therefore, interventions must minimize cost, transportation, and scheduling burdens.

## Strengths of the study

This study provides in-depth insight into an understudied population, family caregivers of chronic stroke survivors living in rural, under-resourced settings. Methodological rigor was enhanced through investigator triangulation, iterative analysis, and the use of direct participant quotations, strengthening the credibility of findings (31).

## Limitations of the study

Findings may have limited transferability beyond similar rural contexts. Participants were self-selected and may overrepresent caregivers who are more engaged or motivated. This qualitative design priorities depth of experience rather than generalizability. This study was conducted in rural, under-resourced settings, which may limit generalizability to urban or higher-resource contexts. Participants were self-selected and may overrepresent caregivers who are motivated or technologically engaged. Data were self-reported, introducing potential recall or social desirability bias. Finally, including caregivers of chronic stroke patients may enhance transferability but could reduce the specificity of some findings to stroke care.

## Implications for intervention design

Findings indicate that caregivers value practical, accessible, and low-burden support rather than traditional formal programs. Participants emphasized the need for clear step-by-step guidance delivered through brief videos, telehealth encounters, or digital tools that accommodate financial and transportation constraints (10) Interventions that align with caregivers’ real-world needs and existing coping strategies may enhance engagement and sustainability. (11, 27, 32, 33).

## Conclusion

Family caregivers of stroke survivors demonstrate strong intrinsic motivation, adaptability, however, participant accounts clearly illustrate ongoing structural barriers, including limited access to formal support and reliance on informal learning strategies. In the absence of accessible services, caregivers depend on informal learning, digital resources, and family networks to sustain care. These findings highlight a gap between caregiver needs and existing support models. As reflected in caregivers’ reliance on self-directed strategies. Addressing this gap requires affordable, accessible, and family-centered interventions that deliver practical, skills-based guidance through intuitive digital and telehealth platforms. Aligning caregiver support with real-world caregiving demands may improve caregiver well-being and competence, and ultimately enhancing stroke recovery and rehabilitation outcomes.

## Data Availability

The raw data supporting the conclusions of this article will be made available by the authors, without undue reservation.
